# Preparation, Characterization and Performance of Templated Silica Membranes in Non-Osmotic Desalination

**DOI:** 10.3390/ma4040845

**Published:** 2011-04-29

**Authors:** Bradley P. Ladewig, Ying Han Tan, Chun Xiang C. Lin, Katharina Ladewig, João C. Diniz da Costa, Simon Smart

**Affiliations:** 1 Department of Chemical Engineering, Faculty of Engineering, Monash University, VIC 3800, Australia; E-Mail: bradley.ladewig@eng.monash.edu.au; 2 ARC Centre for Excellence in Functional Nanomaterials, The University of Queensland, Cooper Rd, Brisbane, QLD 4072, Australia; E-Mail: kladewig@unimelb.edu.au; 3 AIBN—Australian Institute of Bioengineering and Nanomaterials, The University of Queensland, Cooper Rd, Brisbane, QLD 4072, Australia; 4 Films and Inorganic Membrane Laboratory, School of Chemical Engineering, The University of Queensland, Cooper Rd, Brisbane, QLD 4072, Australia; E-Mails: s4112833@student.uq.edu.au (Y.H.T.); cynthia.lin@uq.edu.au (C.X.C.L.); j.dacosta@uq.edu.au (J.C.D.C.)

**Keywords:** desalination, inorganic membranes, silica, carbonized templates, surfactants, salt rejection

## Abstract

In this work we investigate the potential of a polyethylene glycol-polypropylene glycol-polyethylene glycol, tri-block copolymer as a template for a hybrid carbon/silica membrane for use in the non-osmotic desalination of seawater. Silica samples were loaded with varying amounts of tri-block copolymer and calcined in a vacuum to carbonize the template and trap it within the silica matrix. The resultant xerogels were analyzed with FTIR, Thermogravimetric analysis (TGA) and N_2_ sorption techniques, wherein it was determined that template loadings of 10 and 20% produced silica networks with enhanced pore volumes and appropriately sized pores for desalination. Membranes were created via two different routes and tested with feed concentrations of 3, 10 and 35 ppk of NaCl at room temperature employing a transmembrane pressure drop of <1 atm. All membranes demonstrated a salt rejection capacity of >85% (in most cases >95%) and fluxes higher than 1.6 kg m^−2^ h^−1^. Furthermore, the carbonized templated membranes displayed equal or improved performance compared to similarly prepared non-templated silica membranes, with the best results of a flux of 3.7 kg m^−2^ h^−1^ with 98.5% salt rejection capacity, exceeding previous literature reports. In addition, the templated silica membranes exhibited superior hydrostability demonstrating their potential for long-term operation.

## 1. Introduction 

Desalination is an established technology for the supply of potable water to coastal and inland communities. Early large scale desalination was achieved using thermal technologies such as multi-effect distillation (MED) and multi-stage flash distillation (MSF). Nowadays reverse osmosis (RO) is the technology of choice for new installations reflected by 44% of world desalination capacity and 80% of the number of plants [1]. However, many challenges remain in the use of RO for the desalination of seawater, most notably the significant energy consumption of approximately 1.9–4.2 kWh per m^3^ of product water [2], which is mainly associated with high pressure requirements to overcome the osmotic pressure. 

One alternative approach to desalination is pervaporation using inorganic membranes. In this technology, the saline feed is provided at one side of a membrane, while water vapor is removed on the permeate side and condensed accordingly. This approach has been demonstrated for ceramic membranes such as zeolites [3,4,5] and silica [6]. Silica membranes are attractive, as their preparation using sol-gel processes delivers molecular sieving pore size domains, advantageous for desalination applications. On the other hand, silica membranes generally are composed of silanol groups (Si–OH), which are highly hydrophilic and siloxane (Si–O–Si) bonds which can be hydrolyzed in the presence of water. Hence, silica membranes are unstable when exposed to water, undergoing severe structural modification, pore size widening, and loss of separation performance [7,8]. 

In order to overcome this technical shortfall for desalination applications, Duke *et al*. carbonized covalent bonded methyl templates to silica, or ionic C6 surfactants in the silica matrix [6]. This approach was based on the principle that carbonization of organic templates provides superior silica structural hydrostability. It was demonstrated that the carbonization of surfactants gave a higher salt rejection though at lower flux. Subsequently, Wijaya *et al*. investigated the effect of the carbonization of surfactants with varying carbon chain lengths, and reported that carbonized template silica membranes increased their salt rejection from 92 to 94 and 97% by increasing the surfactant’s carbon chain from C6 to C12 and C16, respectively [9]. Hence, these results strongly suggest that the incorporation of carbon in a silica matrix plays a role in salt rejection as well as matrix stabilization. The difficulty here is that ionic templates such as surfactants tend to form micelles at high concentrations [10] and precipitate if in excess of 3 wt% in the silica sol-gel. Thus, the incorporation of carbon into the silica matrix is limited in this case.

In this work, we test the novel idea of increasing the carbon content in the silica matrix by using polymeric templates instead. These are non-ionic templates thus allowing the carbon loading in silica matrix to be increased beyond the previous limitations on ionic surfactants. We describe the synthesis of a new class of silica membranes using tri block co-polymer templates. We demonstrate that in using inorganic membranes and a pervaporation approach, the desalination of brackish water and seawater is possible with only approximately 1 bar pressure difference.

## 2. Results and Discussion 

**Figure 1:**
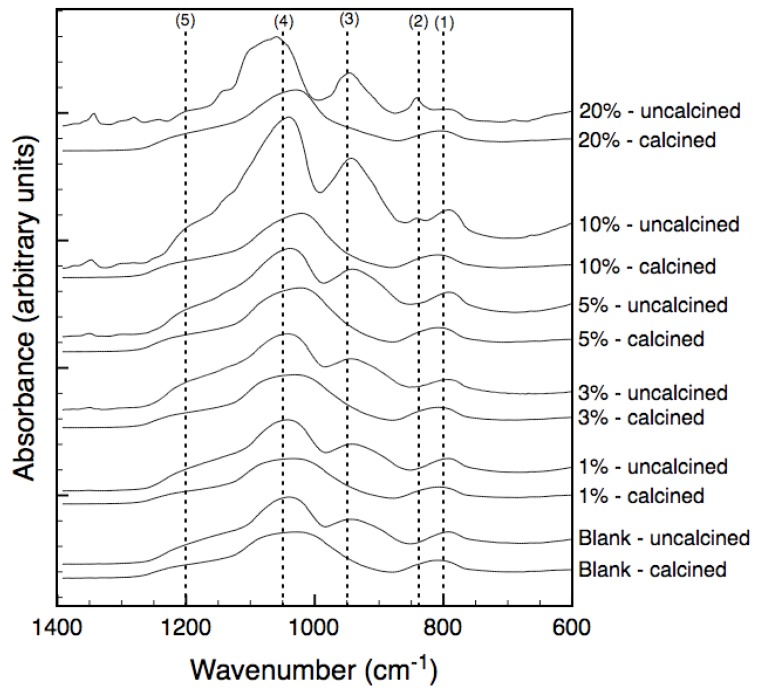
FTIR spectra of bulk xerogels treated at 50 °C (uncalcined) and 600 °C (calcined). Percent quantities refer to the mass percent polymer in the precursor solution.

The FTIR spectra for uncalcined (air dried at 50 °C) and calcined (at 600 °C under vacuum) xerogels are displayed in Figure 1. The bands at 800, 1,050 and 1,200 cm^−1^ correspond to the various stretching modes of siloxane (Si–O–Si) bonds while the band at 950 cm^−1^ is assigned to stretching vibrations of silanol (Si–OH) bonds. Similar spectra are observed for both non-templated and templated xerogels at 50 °C and 600 °C with the major differences being peak broadening of bands at 800 cm^−1^ and between 1,050–1,200 cm^−1^ and the disappearance of the silanol band at 950 cm^−1^ when treated at 600 °C. These findings are in good agreement with the literature [11,12] where polycondensation is the underlying process for decreasing the silanol groups and increasing siloxane groups. An additional band at 840 cm^−1^ was observed in the uncalcined template samples containing 10% and 20% tri-block copolymer and was attributed to the CH_2_ rocking vibrations within the PEG-PPG-PEG template [13]. The similarity between the spectra of the templated and non-templated samples after calcination, even at high loadings, demonstrates that the tri-block copolymer no longer exists in its polymeric form. 

**Figure 2:**
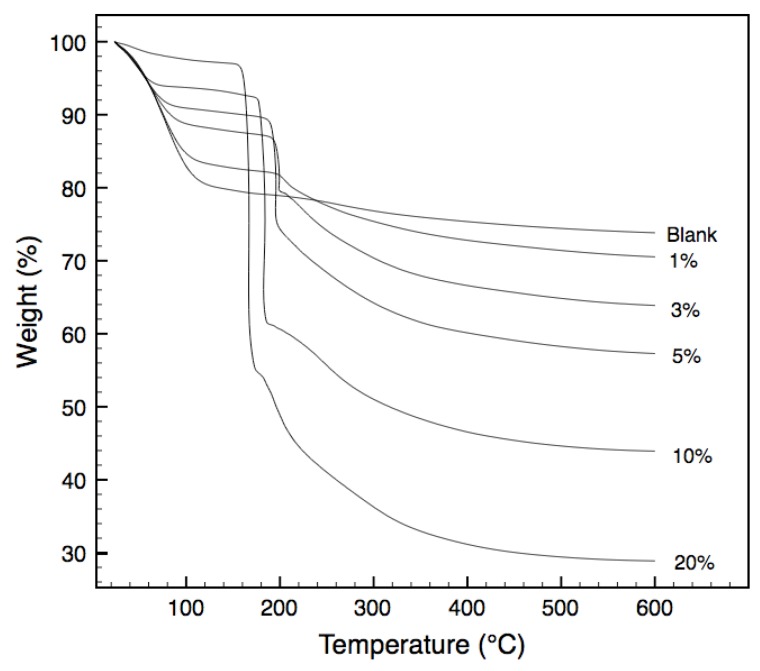
Thermogravimetric weight loss for samples tested under air atmosphere.

Thermogravimetric analysis (TGA) of the non-templated and templated xerogels in air, are presented in Figure 2. Weight losses from room temperature to approximately 120 °C are largely due to the evaporation of water molecules from the xerogel matrix. Subsequent steady weight losses from 120 °C to approximately 200 °C account for the continual expulsion of water and ethanol from the matrix as a result of condensation reactions. Understandably, the behavior of the non-templated xerogels is different to those of the templated ones. Sharp weight losses between 155 °C and 220 °C that are observed in the templated samples do not occur in the non-templated samples. They are mainly attributed to the oxidation of the tri-block copolymer templates embedded in the xerogel matrix [14]. As expected, the weight losses observed in this temperature varies in accordance with the template loading in the xerogel. Less expected was the variation in the temperature at which these oxidation reactions occur, with increased loading resulting in a decreased oxidation temperature. Indeed, loadings above 1% resulted in oxidation temperatures below that of the pure PEG-PPG-PEG tri-block copolymer. Similar trends were observed by Lazzaro and Milioto in 2010, when nanosilica spheres were dispersed through a series of low molecular weight polymers, including PEG-PPG-PEG [15]. The authors determined that the silica surface was acting to enhance the oxidation of the polymer in the composites, at temperatures below that of the pure polymer. Whilst the morphology of Lazzaro and Milioto’s composite differs from this work with silica nanoparticles within a polymeric matrix in contrast to polymer chains trapped within a silica matrix, it is postulated that the catalytic effect of the silica surface to lower the oxidation temperature of the PEG-PPG-PEG polymer remains.

**Figure 3:**
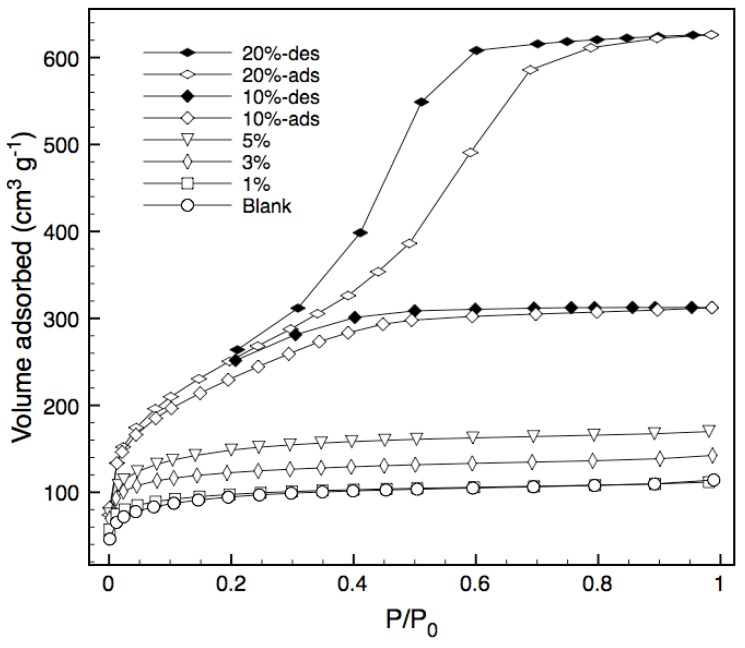
Nitrogen sorption isotherms. The desorption branch is only shown for 10% and 20% samples, as the other samples displayed negligible hysteresis.

Nitrogen adsorption isotherms for various bulk xerogels calcined at 600 °C under vacuum are shown in Figure 3, whilst Table 1 summarizes the structural characteristics of the xerogels, including the Bernard, Emmett and Teller (BET) surface area, total pore volume and average pore radius. Adsorption isotherms for the blank silica and templated xerogels containing 1%, 3% and 5% block copolymer are of Type I and indicate a microporous structure. 

Increasing the loading of the tri-block copolymer to 10% effectively doubles the pore volume and surface area, whilst still maintaining a type I isotherm and thus a high percentage of micropores. Further increases in loading to 20% causes the isotherm to change from Type I to Type IV, indicating the occurrence of capillary condensation [16] and that pore structure of the resultant templated silica is altered from microporous to mesoporous. In general, surface area and total pore volume increase with increasing tri-block copolymer loading, while the average pore radius does not vary except at high concentrations. This increase in surface area can be attributed to the increased amount of pyrolyzed template embedded in the xerogel matrix as carbon [9]. The increase in pore volume associated with increased template loading also suggests an increase in porosity which should result in more permeable structures. The increase in pore radius for the 10 and 20% samples indicates that there is a cut-off point for template loading, around 10%, at which the silica matrix transitions from a microporous to a mesoporous structure. This suggests that some level of pore size and porosity tuning can be obtained through careful selection of template content.

**Table 1:** Microstructural characteristics of bulk xerogels.

Sample	BET surface area (m^2^ g^−1^ ± 10%)	Total Pore Volume (cm^3^ g^−1^ ± 10%)	Average *R_p_* (nm ± 15%)
Blank	334	0.177	1.06
1%	339	0.173	1.02
3%	427	0.220	1.03
5%	519	0.263	1.01
10%	837	0.483	1.15
20%	922	0.969	2.10

**Figure 4:**
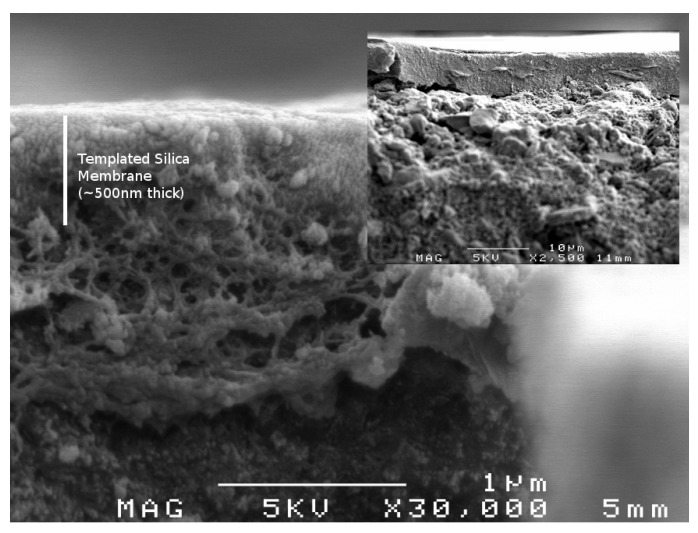
Scanning electron microscopy (SEM) image of membrane with 10% template loading showing close up of the membrane layer, **(inset)** overall hierarchical structure of membrane.

Membrane performance is judged on two main criteria, the flow rate that the membrane can process or flux and the ability of the membrane to separate components or selectivity. For these porous templated silica membranes the pore volume controls the flux whilst the pore radius, through the mechanism of size exclusion, controls the selectivity. Previous investigations have demonstrated that templated silica membranes provide excellent salt rejection but low fluxes in comparison to established technologies [6,9]. As pore volume and pore radius are material characteristics that are inherently linked, there will always be a trade-off between flux and selectivity. However, the greater control over pore radius and pore volume afforded by PEG-PPG-PEG template raised the possibility of high flux templated silica membranes, which would be very attractive for industrial applications. To this end membranes were produced with 10 and 20% template loading and tested under a variety of desalination conditions. A representative scanning electron microscopy (SEM) image of a membrane with 10% template loading is displayed in Figure 4. The chosen compositions offered additional intrigue in that their silica networks were in transition between highly microporous and highly mesoporous structures. 

**Figure 5:**
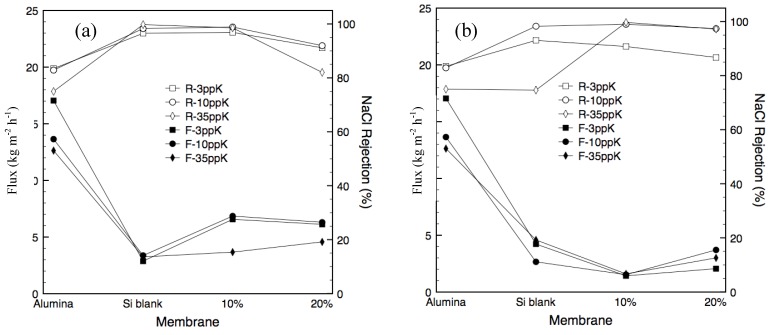
**(a)** Membrane flux and separation for Series 1 **(b)** Membrane flux and separation for Series 2. The hollow symbols denoted ‘R-’ represent salt rejection, whilst the solid symbols denoted ‘F-’ represent membrane flux.

**Figure 6:**
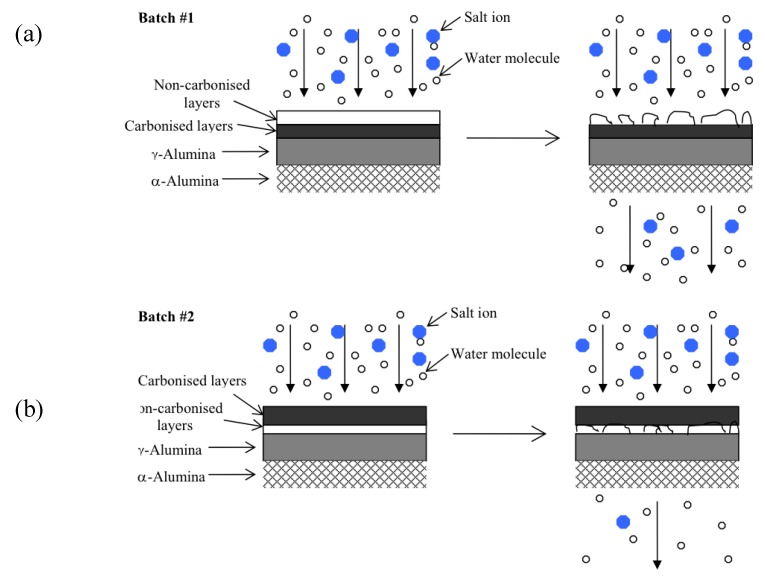
**(a)** Proposed model for membranes from series 1 **(b)** Proposed model for membranes from series 2.

Further to this, membranes with 10% and 20% template loading were synthesized in two series. The first used a predominately oxidative atmosphere in which much of the PEG-PPG-PEG template would have oxidized (as per Figure 2). The second series was calcined under predominately vacuum conditions ensuring more of the PEG-PPG-PEG template was carbonized and remained trapped in the silica matrix. Exact synthesis details are provided in the Experimental section in Table 3 whilst a schematic showing the layered structure is given in Figure 6a. The motivation behind this was to determine the importance of the protective effect of the carbonized template on the structural stability of the silica against the increased pore volume, and thus increased flux, afforded by partial template removal. The flux and salt rejection characteristics for the carbonized templated membranes with 10 and 20% template loading are given in Figure 5a and Figure 5b, respectively. The performance of the alumina support and a non-templated silica membrane are provided for comparison. 

There was a consistent decrease in flux with increasing feed concentration for all membranes tested (including the alumina support) which is consistent with similar studies reported elsewhere [6,9,17,18]. The higher salt concentration results in a decrease of the chemical potential gradient or driving force across the membrane and also serves to the hinder the flow of water molecules to the surface of the membrane through osmotic gradients in the on the feed side. The alumina support had the highest flux as expected given it has the largest pore size and hence the least resistance to flow. It was interesting to observe that the support showed a salt rejection of >70% for a 35 ppk feed, despite there being no selective layer to provide a molecular sieving effect. In principle, the alumina support should act in a similar fashion to inorganic nanofiltration membranes which can desalt water through the pressure driven electrokinetic effect associated with charged porous media [19,20]. However, this result is in direct contrast to a previous study wherein the same alumina substrates showed a negative rejection mechanism, or a positive selection for salt [6]. Hence it is likely that the pH of the salt feed solutions has varied either side of the iso-electric point of the α-alumina substrate in this study and the preceding one. 

The non-templated membrane (blank silica) displayed the lowest flux for series 1 and close to the lowest for series 2. This is consistent with the bulk xerogel nitrogen sorption analysis for the non-templated material, which showed that it had a microporous structure with a pore volume considerably smaller than the 10 and 20% templated materials. More importantly, the flux of the pure silica membrane was similar regardless of the synthesis route. However, the selectivity of the membranes varied significantly between series. The blank silica membrane produced in series 1 consistently rejected more than 95% of the salt in the feed regardless of concentration. In contrast the membrane produced in series 2 exhibited significant degradation during the 35 ppk test. As these tests were performed chronologically with increasing salt concentration this deterioration in performance agrees well with the view held in literature that pure silica is not stable when exposed to water [21]. The results from series 1 seem to contradict previous findings; however the exposure was limited to a total of 12 h testing plus permeate flushing. Thus it is hypothesized that longer exposure times would have resulted in similar performance degradation as observed in the series 2 membrane.

The membranes containing 10 and 20% template loading from series 1 displayed higher fluxes than for series 2, which was expected given partial removal of the carbonized template would have resulted in a larger effective pore size. Likewise the selectivity of the membranes produced in series 2 was, for the most part, higher than for those membranes produced in series 1. The primary difference was with the lowest feed concentration where the salt rejection was 90% or below for the templated membranes produced in series 2, compared to 90% or greater for the templated membranes produced in series 1. It should be noted however, that the actual permeate salt concentrations were <500 ppm regardless of synthesis route, suggesting that the relative experimental error in calculating salt rejection at low feed concentrations is high. 

The 20% PEG-PPG-PEG templated membrane synthesized in series 1 experienced a decline in selectivity as testing time progressed, similar to the blank silica membrane. This is attributed to the reduced amount of carbonized template within the silica matrix and the corresponding reduction in hydrostability. Furthermore, as the oxidation and thus removal of the template would have been greatest in the topmost layers these would have degraded first, as shown in Figure 6a. The degradation of these topmost, predominately pure silica layers would have likely introduced defects into the entire membrane structure resulting in both increased flux and decreased selectivity. By contrast the membranes produced in series 2 had the more stable templated layers exposed to the feed solution and thus responsible for the selectivity of the membrane (Figure 6b). Any pore widening in the underlying silica layers would have had minimal impact on the selectivity of the entire membrane as the separation of water from hydrated salt ions had already taken place.

Finally, Table 2 compares the performance of the membranes in this work to previously reported values in literature. The results clearly indicate that, membranes produced using the PEG-PPG-PEG template are superior to previously reported inorganic membranes. It should also be noted that the testing was conducted with a transmembrane pressure difference of <1 atm at room temperature suggesting that these membranes can operate with minimal energy requirements.

**Table 2:** Experimental results compared to literature values.

Membrane	Flux (kg m^−2^ h^−1^)	NaCl rejection (%)	Feed salinity (ppk)	Reference
10% (series 1)	3.7	98.5	35	-
10% (series 2)	1.6	99.7	35	-
20% (series 2)	3.0	97	35	-
CTMSS (C16)	2.2	97	35	[9]
CTMSS	1.9	98	35	[6]
CTMSS—after regeneration	1.5	99	35	[6]
Zeolites	0.12	77	6	[4]

## 3. Experimental Section 

We employed a two-step acid catalyzed hydrolysis sol-gel method described elsewhere [22]. Briefly, tetraethylorthosilicate (TEOS) and ethanol (EtOH) were initially mixed in an ice bath to prevent premature hydrolysis [23]. Addition of dilute acid was then conducted dropwise with constant stirring to produce a solution with an initial molar ratio of 1.0 TEOS: 3.8 EtOH: 1.0 H_2_O: 7 × 10^−4^ 1M HNO_3_. The solution was refluxed with constant stirring at 60 °C for 90 minutes and subsequently cooled to room temperature. Then, additional 1 M HNO_3_ and water were added to give a final molar composition of 1.0 TEOS: 3.8 EtOH: 6.0 H_2_O: 0.1 1M HNO_3_. The templated sol was prepared as described for the two-step sol, with the addition of PEG-PPG-PEG, a tri-block copolymer (Sigma Aldrich), in different amounts to produce mixtures with Si:copolymer ratios of 1, 3, 5, 10 and 20 wt%. The required mass of copolymer was added to the as-prepared two-step sol and the reaction mixture was shaken continuously until the copolymer completely dissolved.

Xerogel samples obtained from an identical sol-gel synthesis process were heated to 600 °C under vacuum at 1 °C/min, held for four hours and then cooled at 1 °C/min to room temperature. The inert vacuum conditions allowed for the copolymer template to be carbonized within the silica matrix, using similar procedures as the membrane calcination process. The xerogel samples were characterized using a Nicolet 6700 FT-IR instrument for infrared spectroscopy, a Shimadzu TGA-50 at a heating rate of 5 °C/min for thermogravemetric analysis and a Quantachrome NOVA-1200 instrument for sorption analysis. 

Substrates used were α-alumina with 30% porosity, average pore size of 0.5–1.0 μm and 2 mm thickness (Rojan Advanced Ceramics Pty Ltd). The surface to be treated was sanded with 1200 and then 2000 grade sandpaper, before being ultrasonicated for 1–2 min in a water bath to remove any loose powder. The substrates were then coated with intermediate layers of boehmite sol. Coating was accomplished using an automated dip-coater housed in a class 100 laminar flow cabinet (to minimize defect formation from adhered dust particles). Both blank silica and templated silica sols were diluted 1:19 by weight with ethanol prior to film deposition. The coating conditions for both the intermediate and membrane layers are summarized in Table 3. 

**Table 3:** Synthesis conditions for various membrane layers.

Series	Layer	Atmosphere	No. of Coats	Calcination Temperature (°C)	Immersion Time (min)	Dipping Speed (cm/min)
1	Intermediate (boehmite sol)	Air	2	600	0	2
	Silica/Templated Silica	Vacuum	3	600	1	2
		Air	3	600	1	2
2	Intermediate (boehmite sol)	Air	2	600	0	2
	Silica/Templated Silica	Air	2	600	1	2
		Vacuum	4	600	1	2

**Figure 7:**
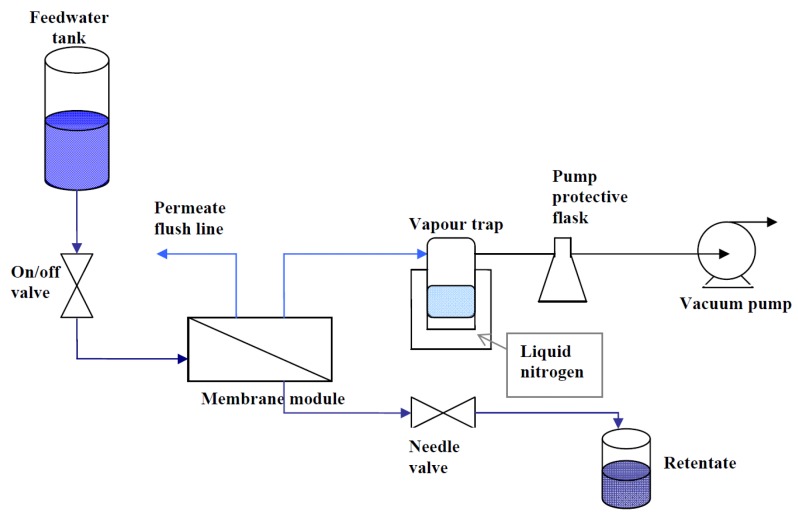
Schematic of the pervaporation system used to test the supported membranes.

The supported membranes were inserted into a Teflon membrane module with the coated membrane side contacted by the feed solution and the uncoated substrate facing the permeate side. The module was connected to a pervaporation system as depicted in Figure 7. Feed solutions were prepared by dissolving laboratory grade NaCl (Sigma Aldrich) in deionized water, to produce 3, 10 and 35 ppk solutions. A labCHEM CP conductivity meter was used to measure the conductivities of the feed, retentate and permeate solutions. The conductivity meter was calibrated with standard NaCl solutions to produce a conductivity *vs*. concentration curve. The retentate valve was adjusted to achieve a constant flow of approximately 3.3 mL min^−1^, to minimize concentration polarization at the membrane surface. The permeate water vapor was removed from the permeate side and was collected in a cold trap filled with liquid nitrogen. The feed tank was open to the atmosphere, so the pressure drop across the membrane was approximately 1 atm for all measurements. 

The mass flux, *F* (kg m^−2^ h^−1^) through the membranes was calculated according to the equation *F = m/(At)*, where *m* is the mass of permeate (kg) measure from weighing the water retained in the cold trap, *A* is the membrane active area (1.02 × 10^−4^ m^2^) and *t* is the measurement duration (h). The membrane salt rejection, *R* (%), was calculated as *R = (C_feed_ − C_p_)/C_feed_ × 100%*, where *C_feed_* and *C_p_* are the feed and permeate concentrations (ppk), respectively. 

## 4. Conclusions 

Templated molecular sieve silica membranes were prepared on α-alumina substrates with boehmite intermediate layers, and calcined under vacuum to carbonize the template and trap it within the silica matrix. As-prepared membranes demonstrated considerable potential for the desalination of synthetic seawater (35 ppk NaCl solution), achieving a best result of a flux of 3.7 kg m^−2^ h^−1^ with 98.5% rejection at room temperature employing a transmembrane pressure drop of <1 atm. The carbonized, templated membranes displayed equal or higher fluxes than similarly prepared non-templated silica membranes, and dramatically increased salt rejection over the uncoated α-alumina substrates. These results, taken in conjunction with the observed superior hydrostability, demonstrate their potential for long-term industrial operations. 
